# Active and passive physical therapy in patients with chronic low-back pain: a level I Bayesian network meta-analysis

**DOI:** 10.1186/s10195-025-00885-4

**Published:** 2025-10-03

**Authors:** Alice Baroncini, Nicola Maffulli, Nicola Manocchio, Michela Bossa, Calogero Foti, Luise Schäfer, Alexandra Klimuch, Filippo Migliorini

**Affiliations:** 1 Department of Spine Surgery, Casa di Cura Humanitas San Pio X, Milan, Italy; 2 Faculty of Medicine and Psychology, University “La Sapienza” of Rome, Rome, Italy; 3 Faculty of Medicine, School of Pharmacy and Bioengineering, Keele University, Stoke on Trent, ST4 7QB UK; 4 Centre for Sports and Exercise Medicine, Barts and the London School of Medicine and Dentistry, Mile End Hospital, Queen Mary University of London, London, E1 4DG UK; 5 Physical and Rehabilitation Medicine, Clinical Sciences and Translational Medicine Department, Tor Vergata University, Rome, Italy; 6 Department of Orthopaedic and Trauma Surgery, Eifelklinik St. Brigida, Kammerbruschstr. 8, Simmerath, 52152 Aachen, Germany; 7 Department of Trauma and Reconstructive Surgery, University Hospital of Halle, Martin-Luther University Halle-Wittenberg, Ernst-Grube-Street 40, 06097 Halle (Saale), Germany; 8 Department of Orthopaedic and Trauma Surgery, Academic Hospital of Bolzano (SABES-ASDAA), Via Lorenz Böhler 5, 39100 Bolzano, Italy; 9 Department of Life Sciences, Health, and Health Professions, Link Campus University, Via del Casale di San Pio V, 00165 Rome, Italy; 10 PhD Course in Tissue Engineering and Remodeling Biotechnologies for Body Function, Clinical Sciences and Translational Medicine Department, Tor Vergata University, Rome, Italy; 11 Department of Orthopaedic, Trauma and Reconstructive Surgery, RWTH Aachen University Hospital, Aachen, Germany

**Keywords:** Spine, Low back pain, Physiotherapy

## Abstract

**Background:**

Chronic low back pain (cLBP) is common. Physiotherapy is frequently indicated as a non-pharmacological management of these patients. This Bayesian network meta-analysis compared active versus passive physiotherapy versus their combination in terms of pain and disability in patients with mechanical and/or aspecific cLBP.

**Methods:**

In June 2025, the following databases were accessed: PubMed, Web of Science, Google Scholar and Embase. All the randomised controlled trials (RCTs) which evaluated the efficacy of a physiotherapy program in patients with LBP were accessed. Data regarding pain scores, the Roland–Morris Disability Questionnaire (RMQ) and the Oswestry Disability Index (ODI) were collected. The network meta-analyses were performed using the STATA (version 14; StataCorp, College Station, TX, USA) routine for Bayesian hierarchical random-effects model analysis, employing the inverse variance method. The standardised mean difference (STD) was used for continuous data.

**Results:**

Data from 2768 patients (mean age 46.9 ± 10.9 years, mean BMI 25.8 ± 2.9 kg/m^2^) were collected. The mean length of follow-up was 6.2 ± 6.1 months. Between groups, comparability was found at baseline in terms of mean age, proportion of women, mean BMI, symptom duration and patient-reported outcome measures (PROMs). By the end of the follow-up period, the active group evidenced the lowest pain scores (SMD 1.00; 95% CI −3.28 to 5.28). The active group evidenced the lowest RMQ score (SMD 0.94; 95% CI –4.96 to 3.09). The active group evidenced the lowest ODI score (SMD −1.23; 95% CI −9.83 to 7.36).

**Conclusion:**

Active physiotherapy showed better results than passive physiotherapy and a combination of both for the management of mechanical and/or non-specific cLBP.

*Level of evidence*: Level I, Bayesian network meta-analysis of RCTs.

## Introduction

Chronic low back pain (cLBP) is a significant cause of disability and health care expenditure worldwide [1, 2]. Current guidelines suggest that non-pharmacological treatment should be the first measure to adopt in the management of chronic low back pain (cLBP), followed by pharmacological therapy in cases of non-pharmacological treatment failure [3, 4]. Among the various available options, physiotherapy represents one of the most widely used first-line treatments for cLBP [5–7]. Different physiotherapeutic interventions have been investigated to assess their efficacy in improving painful symptoms and disability [8, 9]. However, the heterogeneity of treatment types and protocols makes it problematic to group the available randomised controlled trials (RCTs) to obtain strong evidence supporting any given treatment.

Physiotherapeutic regimes can be broadly categorised as active, in which patients are prompted to perform exercises to improve their mobility and strength [10, 11], or passive, where the patient receives a treatment (e.g. massages or joints mobilizations) without actively engaging in physical activity [12, 13]. A combination of both techniques is also possible. To maximise the available data on the outcomes of physiotherapeutic management in the setting of cLBP, and to allow for a direct comparison, the present work categorised the available techniques and regimens into three groups: active, passive and combined physiotherapy. A Bayesian network meta-analysis was then conducted to compare these three options, aiming to identify which one is most effective in terms of pain and disability improvement in the non-pharmacological management of mechanical and aspecific cLBP.

## Methods

### Eligibility criteria

All the randomised controlled trials (RCTs) which evaluated the efficacy of a physiotherapy program in patients with LBP were accessed. According to the authors’ language capabilities, articles in English, German, Italian, French and Spanish were eligible. Only RCTs with level I of evidence, according to the Oxford Centre of Evidence-Based Medicine [14], were considered. Reviews, opinions, letters and editorials were not considered. Animal, in vitro, biomechanics, computational and cadaveric studies were not eligible. Studies reporting on non-specific [15] or mechanical [16] cLBP were included. The pain was defined as chronic when symptoms persisted for a minimum of 3 months [17]. Studies including patients with radiculopathy and/or neurologic symptoms were excluded from this analysis. Missing quantitative data on the outcomes of interest warranted the exclusion of the study.

### Search strategy

This study was conducted in accordance with the 2015 PRISMA Extension Statement for Reporting of Systematic Reviews Incorporating Network Meta-Analyses of Health Care Interventions [18]. The following algorithm was established:

- P (problem): cLBP
- I (intervention): physiotherapy
- C (comparison): active versus passive physiotherapy versus a combination of both
- O (outcomes): pain and disability

In June 2025, the following databases were accessed: PubMed, Web of Science, Google Scholar and Embase. No time constraint was set for the search. The search was restricted to only RCTs. The matrix of keywords used in each database is shown in the Appendix. No additional filters were used in the database search.

### Selection and data collection

Two authors (A.K. and L.S.) independently performed the database search. All the resulting titles were screened by hand, and if suitable, the abstract was accessed. The full text of the abstracts which matched the topic was accessed. If the full text was not accessible or available, the article was excluded from consideration. A cross-reference of the bibliography of the full text was also conducted to identify additional studies. Disagreements were settled by a third author (N.M.).

### Data categorisation

Data categorisation was conducted by three experienced physiatrists (F.C., B.M. and M.N.). In the field of physiotherapy, a fundamental distinction exists between active and passive interventions. Active physiotherapy involves the active participation of the patient in performing therapeutic exercises or activities that promote mobility, strength and functional improvement [19]. This intervention encourages patients to take an active role in their rehabilitation, fostering self-management and independence [20]. On the other hand, passive physiotherapy refers to interventions where the patient receives treatment without actively engaging in physical movements, such as manual therapy techniques, kinesiotaping or modalities such as heat or electrical stimulation. It relies on external therapeutic interventions facilitated by the physiotherapist on the affected muscles, which often appear hypercontracted [21, 22]. Passive stretch reduces stiffness (viscoelastic stress relaxation) and decreases stretch-induced pain. This transient reduction in stiffness may persist for 1–2 h before returning to pre-stretch levels [23, 24]. Moreover, daily passive stretching (15–60 s) reduces muscle stiffness over the following 24 h [25]. The criteria for categorising interventions into active or passive modalities include the level of patient effort, the type of movement involved and the extent of therapeutic guidance provided by the physiotherapist [26]. Active physiotherapy often requires patients to exert voluntary effort and participate in active movements aimed at restoring function and improving physical capacity [27]. Passive physiotherapy, on the other hand, focusses on the therapist’s direct application of techniques to the patient [26]. Finally, in terms of pain relief and/or recovery in activities of daily living, passive treatment can help with immediate pain relief, but active treatment keeps the patient functional in the long term. For that reason, many passive interventions have shown positive effects for acute LBP [28, 29].

### Data items

Two authors (A.K. and L.S.) independently performed data extraction. The following data at baseline were extracted: author and year of publication, journal of publication, men:women ratio, number of patients included with related mean age and BMI (kg/m^2^), mean length of symptoms duration prior to the physiotherapy and the length of the follow-up. Data concerning the following PROMs were collected at baseline and at the last follow-up: pain scores, Roland–Morris Disability Questionnaire (RMQ) [30] and Oswestry Disability Index (ODI) [31]. To evaluate the pain scores, a visual analogue scale (VAS) or numeric rating scale (NRS) was used. As VAS and NRS showed a high correlation, these were used interchangeably for the present work [32]. Data were extracted in Microsoft Office Excel version 16.72 (Microsoft Corporation, Redmond, USA).

### Assessment of the risk of bias and quality of the recommendations

The risk of bias of the included RCTs was evaluated in accordance with the recommendations of the Cochrane Handbook for Systematic Reviews of Interventions [33]. Two reviewers (A.K. and L.S.) independently assessed all studies, and any disagreement was resolved by discussion with a third senior author (N.M.). The following domains were evaluated: selection bias (random sequence generation and allocation concealment), performance bias (blinding of participants and personnel), detection bias (blinding of outcome assessors), attrition bias (incomplete outcome data), reporting bias (selective outcome reporting) and other sources of bias. Each domain was rated as low, unclear or high risk of bias, and a summary assessment was provided for each study. This procedure ensured a transparent appraisal of the methodological quality of the available evidence. The overall quality of the recommendations was further interpreted in light of the risk of bias distribution, sample size and consistency of findings across studies, providing a contextual framework for the reliability of the results.

### Synthesis methods

The statistical analyses were conducted by the main author (F.M.) following the methodological guidance of the Cochrane Handbook for Systematic Reviews of Interventions [34]. Descriptive statistics were performed using IBM SPSS version 25, with the mean and standard deviation calculated for continuous variables. Normality of data distribution was verified using the Shapiro–Wilk test, and baseline comparability between groups was assessed with analysis of variance (ANOVA) for parametric data and the Kruskal–Wallis test for non-parametric data, with *P* values greater than 0.1 considered satisfactory. The network meta-analyses were performed in STATA software/MP (version 14; StataCorp, College Station, Texas, USA) using the Bayesian hierarchical random-effects model and the inverse variance method, which represent standard approaches for this type of analysis. The standardised mean difference (SMD) was used for continuous outcomes, with both 95% confidence intervals (CI) and 95% percentile intervals (PrI) reported. To assess the assumption of transitivity, the included studies were carefully evaluated for similarity in design, patient characteristics, interventions and outcome definitions, ensuring that indirect comparisons were clinically meaningful. Statistical inconsistency was assessed using the global Wald test for linearity; if *P*_Wald_ > 0.1, the null hypothesis of consistency could not be rejected, indicating that the direct and indirect estimates were coherent. Heterogeneity across studies was explored through the random-effects model, which accounts for between-study variance. Edge plots were used to visualise direct and indirect comparisons and their relative weights, while interval plots ranked treatments according to estimated effect sizes. To investigate the presence of small-study effects and potential publication bias, funnel plots were generated for each outcome. Greater asymmetry in these plots was interpreted as an indication of increased heterogeneity or bias. Taken together, these analyses allowed a comprehensive assessment of the robustness, validity and reliability of the evidence across the treatment network.

## Results

### Study selection

**Fig. 1:**
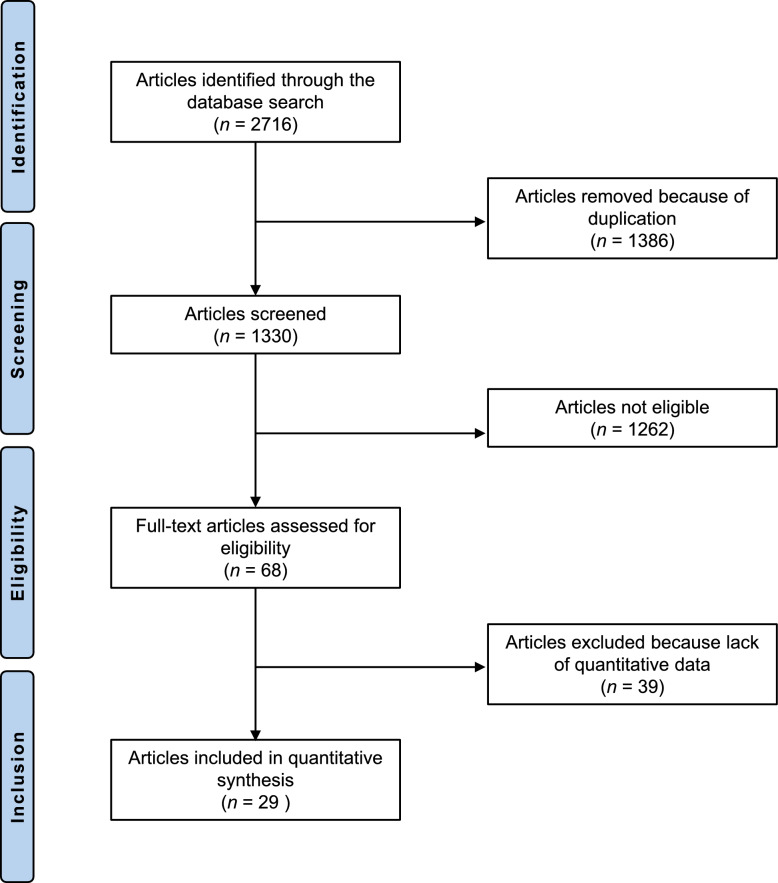
PRISMA flow chart of the literature search

The systematic literature search identified 2716 articles. A total of 1386 were excluded because they were duplicates. Another 1262 articles did not fulfil the eligibility criteria and were therefore excluded. Reasons for non-admission included, in detail, study design (*N* = 950), low level of evidence (*N* = 132), therapy protocols that could not be classified into one of the three groups of interest (*N* = 128) and language limitations (*N* = 20). After full-text evaluation, an additional 39 investigations were excluded because quantitative data on the outcomes of interest were not available. Finally, 29 randomised control trials were available for inclusion. The results of the literature search are shown in Fig. 1.

### Risk of bias assessment

The risk of bias analysis indicated a low risk of selection bias because all studies included were RCTs. A large number of authors reported high-quality allocation concealment, resulting in a low to moderate risk of allocation bias. The lack of information on the blinding of investigators and patients during treatment and follow-up in most studies resulted in a moderate risk of detection and performance bias. Incomplete data due to study dropout during study enrolment or analysis occurred in a few numbers of the included studies, resulting in moderate attrition bias. Reporting bias was low to moderate, and the risk of other biases was low in most studies. In summary, the quality of the methodological assessment was good (Table 1).

**Table 1:** Cochrane risk of bias tool

Author and year	Randomisation	Allocation	Performance	Detection	Reporting	Attrition	Others
Bi et al., 2013 [35]	Low	Low	Unclear	Unclear	Low	Unclear	Unclear
Branchini et al., 2015 [36]	Low	Low	Unclear	Unclear	Low	Unclear	Low
Branchini et al., 2015 [36]	Low	Unclear	High	Low	Unclear	High	Low
Cecchi et al., 2010 [37]	Low	Low	Unclear	Unclear	Low	Unclear	Low
Bronfort et al., 2011 [38]	Low	Low	High	Low	Unclear	High	Low
Cruz-Díaz et al., 2015 [39]	Low	High	Low	Unclear	Unclear	Unclear	Low
Elgendy et al., 2022 [40]	Low	Low	High	Low	Unclear	Low	Low
Cecchi et al., 2010 [37]	Low	Low	Low	Unclear	Low	Low	Low
Garcia et al., 2018 [41]	Low	Low	Low	Low	Low	Low	Low
Goldby et al., 2006 [10]	Low	Low	Low	Unclear	High	Unclear	Unclear
Goldby et al., 2006 [10]	Low	Low	Unclear	Unclear	Low	Low	Low
Costa et al., 2009 [42]	Low	Low	Unclear	Low	High	Unclear	Low
Hicks et al., 2016 [43]	Low	Low	Low	High	Unclear	Low	Low
Cruz-Díaz et al., 2015 [39]	Low	Unclear	Low	Low	Low	Low	Low
Jousset et al., 2004 [44]	Low	Unclear	Unclear	High	Low	Unclear	Low
Elgendy et al., 2022 [40]	Low	Unclear	Low	Low	Low	Low	Low
Mannion et al., 2001 [45]	Low	Low	Low	Low	Low	Unclear	Low
Fukuda et al., 2021 [46]	Low	Low	High	Unclear	Unclear	Low	Low
Marshall et al., 2008 [47]	Low	Unclear	Low	Low	Low	Low	Low
Garcia et al., 2018 [41]	Low	Low	Unclear	Low	Low	Unclear	Low
Monticone et al., 2013 [48]	Low	Low	Low	Unclear	Unclear	Low	Low
Goldby et al., 2006 [10]	Low	Unclear	Unclear	Unclear	Low	High	Low
Murtezani et al., 2011 [49]	Low	Low	Low	Low	Unclear	Low	Unclear
O’Keeffe et al., 2020 [50]	Low	Low	High	Low	Low	Low	Low
Murtezani et al., 2015 [11]	Low	Unclear	Low	Unclear	High	Unclear	Low
Sahin et al., 2018 [13]	Low	Low	Unclear	Low	Low	Unclear	Low
Hernandez-Reif et al., 2001 [51]	Low	Low	Low	Unclear	Low	High	Low
Vibe Fersum et al., 2013 [52]	Low	Unclear	Unclear	Unclear	Unclear	Low	Unclear
Hicks et al., 2016 [43]	Low	Low	Low	Low	Low	Low	Low

To assess the risk of selection bias, the quality of random sequence generation and concealment during patient allocation was examined. The type of blinding during outcome assessment yielded the risk of detection bias. To survey the risk of attrition bias, studies were assessed for incomplete outcome data, such as missing outcome data due to study discontinuation during study enrolment or analysis. Selective publication of results based on their statistical or clinical relevance led to the risk of reporting bias. If authors identified additional risks of bias, these were considered “other biases”

### Study characteristics and results of individual studies

Data from 2768 patients were collected. The mean length of follow-up was 6.2 ± 6.1 months. The mean age was 46.9 ± 10.9 years. The mean BMI was 25.8 ± 2.9 kg/m^2^. The generalities and demographic of the included studies are presented in Table 2.

**Table 2:** Generalities and patient baseline of the included studies

Author, year	Journal	Group	Type of movement	Type of treatment	Patients (*n*)	Mean follow-up (months)	Mean age	Women (%)
Bi et al., 2013 [35]	*Int J Med Res*	Active	Contraction	Pelvic exercise	23	0	29.1	44
		Act & pass	Strengthening	US, short-wave diathermy & strengthening	24		30.9	46
Branchini et al., 2015 [36]	*F1000research*	Act & pass	Pressure	Manual therapy and fascial manipulation	11	3	48.0	64
		Active	Individualised	Respiratory reeducation, propioception, stretching, core stability	13		44.0	69
Bronfort et al., 2011 [38]	*Spine J*	Active	Various	Education & simple exercises	101	9	45.6	58.4
		Passive	High-velocity, low-amplitude	Spinal manipulation	100		45.2	66
		Active	Strengthening	Strengthening	100		44.5	57
Cecchi et al., 2010 [37]	*Clin Rehabil*	Active	Individualised	Back school	68	12	57.9	70
		Act & pass	Individualised	Mobilisation, active exercise, massage of the soft tissues, proprioceptive	68		60.5	61
				Neuromuscular facilitation				
		Passive	Mobilisation, manipulation	Spinal manipulation	69		58.1	69
Costa et al., 2009 [42]	*Phys Ther*	Active	Individualised	Motor control exercise	77	10	54.6	58
		Passive		Detuned US and detuned short-wave	77		52.8	62
Cruz-Díaz et al., 2015 [39]	*Disabil Rehabil*	Act & pass	Individualised	Strengthening	53	11	69.6	100
		Passive	Various	TENS	48		72.7	100
Elgendy et al., 2022 [40]	*Ortop Traumatol Rehabil*	Act & pass	Various	Shock waves	15	0	32.7	
		Active	Stretching, strengthening	Stretching, strengthening exercises	15		33.3	
Fukuda et al., 2021 [46]	*Braz J Phys Ther*	Passive	Mobilisation	Manual therapy, lumbar stabilisation	35	12	35.2	53
		Act & pass	Mobilisation, strengthening	Manual therapy, lumbar stabilisation	35		40.2	53
Garcia et al., 2018 [41]	*BMJ*	Active	Various	McKenzie	74	11	57.5	78
		Passive	Control group	Detuned pulsed ultrasound	73		55.5	74
Goldby et al., 2006 [10]	*Spine*	Active	Stabilisation	Spinal stabilisation & back school	35	24	43.4	68
		Passive	Individualised	Spinal manipulation & back school	37		41.0	70
		Control group	Control	Back school	19		41.5	68
Gwon et al., 2020 [53]	*Physiother Theory Pract*	Act & pass	Side bridge	Vibration & side-lying bridge exercise	15	0	21.9	2
		Active	Side bridge	Side-lying bridge exercise	15		21.6	2
Hernandez-Reif et al., 2001 [51]	*Int J Neuroscience*	Passive	Various	Manual therapy	24	0	43.8	58
		Active	Various	Muscle relaxation exercise			36.7	50
Hicks et al., 2016 [43]	*Clin J Pain*	Passive	Various	Moist heat treatment & US	31	3	69.5	52
		Active	Stabilisation	Trunk training with neuromuscular stimulation	26		70.7	58
Huber et al., 2019 [54]	*BMC Musculoskelet Disord*	Active	Walking	Guided hiking in mountains	27	14	52.9	52
		Act & pass	Walking, heat	Balneotherapy	26		53.4	54
		Control	Control	No intervention	27		43.8	63
Jousset et al., 2004 [44]	*Spine*	Act & pass	Various	Multimodal	43	5	41.4	30
		Active	Individualised	Active exercises	41		39.4	37
Koldaş Doğan et al., 2008 [55]	*Clin Rheumatol*	Active	Walking	Aerobic exercises	19	1	37.1	79
		Act & pass	Various	Hot packs, US, TENS	18		41.5	78
		Control group	Control	Mobilisation and stretching	18		42.1	78
Mannion et al., 1999 [27]	*Spine*	Act & pass	Various	Isometric exercises	46	6	46.3	61
		Active	Low-impact	Stretching and aerobic and muscle-toning exercises	47		45.2	54
		Passive	Various	Physical agents	44		43.7	55
Mannion et al., 2001 [45]	*Spine*	Active	Various	Strengthening, coordination, aerobic	44	12	46.3	61
		Active	Low-impact	Stretching, aerobic, muscle-toning	43		45.2	54
		Passive	Various	Physical therapy	40		43.7	55
Marshall et al., 2008 [47]	*Spine*	Passive	High velocity, low amplitude, various	Isometric then concentric/excentric exercises	12	9	34.3	50
		Passive	High velocity, low amplitude	Manipulation	13		35.8	54
		Active	Non-thrust, various	Abdominal stabilisation	12		33.9	50
		Active	Non thrust	Education	13		41.7	42
Monticone et al., 2013 [48]	*Clin J Pain*	Act & pass	Various	Cognitive–behavioural	45	12	49.0	60
		Active	Various	Mobilisations, stretching, strengthening, postural control	45		49.7	56
Monticone et al., 2014 [56]	*Eur Spine J*	Active	Stabilising	Spinal stabilisation	10	3	58.9	70
		Act & pass	Various	Spinal mobilisation, stretching, strengthening, postural control	10		56.6	40
Murtezani et al., 2011 [49]	*Eur J Phys Rehabil Med*	Active	Individualised	High-intensity aerobics exercise	50	0	51.4	48
		Passive	Various	IFC, TENS, ultrasound, heat	51			49
Murtezani et al., 2015 [11]	*J Back Musculoskelet Rehabil*	Active	Symptom guided	McKenzie	110	3	48.8	25
		Passive	Various	Interferential current, US, and heat	109		47.5	62
O’Keeffe et al., 2020 [50]	*J Sports Med*	Passive	Individualised	Cognitive functional therapy	106	10 to 11	47.0	77.4
		Active	Various	Exercises, relaxation, pain education	100		50.6	70.0
Ozsoy et al., 2019	*Dove Med Press*	Active	Stabilisation	Core stability	21	0	68.1	29
		Act & pass	Various	Core stability, myofascial release	21		68.0	31
Sahin et al., 2018 [13]	*Turk J Phys Med Rehab*	Act & pass	Various	PT (hot pack, US, TENS) & exercise (strengthening and stretching)	50	12	50.4	64
		Active	Stretching & strengthening	Active isotonic and isometric strengthening & stretching	50		46.2	62
Trapp et al., 2015 [57]	*J Back Musculoskelet Rehabil*	Active	Feedback	Exercises with biofeedback	15	0	45.5	33
		Act & pass	Various	Exercises & walking	15		40.6	40
Vibe Fersum et al., 2013 [52]	*Eur J Pain*	Passive	Mobilisation	Mobilisation, manipulation	43	0	42.9	49
		Active	Unknown	Cognitive-functional	51		41.0	53
Yeung et al., 2003 [12]	*J Altern Complement Med*	Active	Various	Warm up and stretching	26	3	55.6	81
		Act & pass	Various	Exercises & electroacupunture	26		50.4	85

RCT, randomised controlled trial; US, ultrasound; TENS, transcutaneous electrical nerve stimulation; IFC, interferential current

### Baseline comparability

Between groups, comparability was found at baseline in mean age, women, mean BMI, duration of symptoms and PROMs (Table 3).

**Table 3:** Baseline comparability

Endpoint	Active(*N* = 1271)	Passive(*N* = 912)	Active & passive(*N* = 521)	*P*
Mean age	46.6 ± 10.6	48.1 ± 11.3	47.2 ± 12.6	0.9
Women (%)	54.1 ± 17.4	62.1 ± 13.2	54.4 ± 24.8	0.3
Mean BMI (kg/m^2^)	26.0 ± 3.2	25.8 ± 2.9	25.6 ± 3.2	0.9
Symptoms (months)	58.6 ± 38.8	73.6 ± 51.2	35.0 ± 36.4	0.1
Pain scores	5.3 ± 1.1	5.8 ± 0.9	5.3 ± 1.3	0.3
RMQ	10.4 ± 2.9	10.0 ± 2.8	10.2 ± 2.9	0.9
ODI	35.4 ± 10.7	30.8 ± 6.3	37.8 ± 16.9	0.5

### Pain scores

**Fig. 2:**
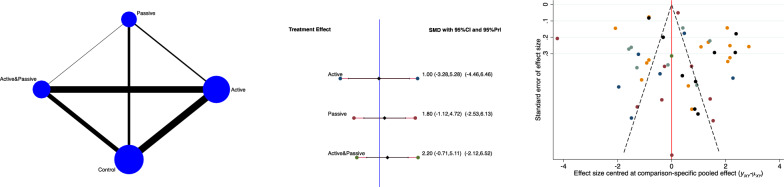
From left to right: edge, interval, and funnel plot of the comparison of pain scores

The active group evidenced the lowest pain scores (SMD 1.00; 95% CI −3.28 to 5.28). The equation for global linearity found no statistically significant inconsistency (*P*_Wald_ = 0.8). These results are shown in Fig. 2.

### RMQ

**Fig. 3:**
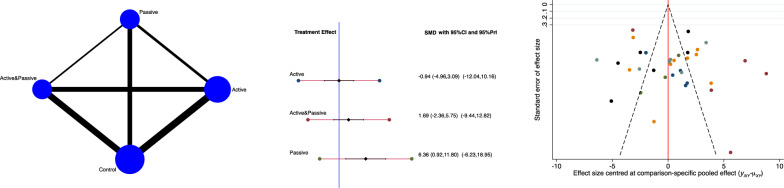
From left to right: edge, interval, and funnel plot of the comparison of RMQ

The active group evidenced the lowest RMQ score (SMD 0.94; 95% CI −4.96 to 3.09). The equation for global linearity found no statistically significant inconsistency (*P*_Wald_ = 0.2). These results are shown in Fig. 3.

### ODI

**Fig. 4:**
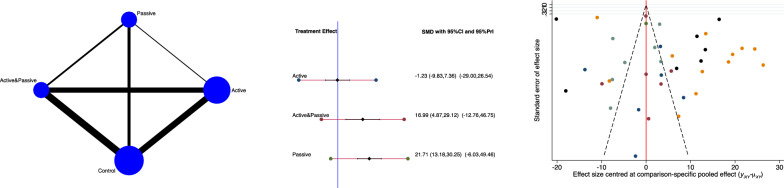
From left to right: edge, interval, and funnel plot of the comparison of ODI

The active group evidenced the lowest ODI score (SMD −1.23; 95% CI −9.83 to 7.36). The equation for global linearity found no statistically significant inconsistency (*P*_Wald_ = 0.6). These results are shown in Fig. 4.

## Discussion

Chronic low back pain represents one of the most prevalent musculoskeletal disorders worldwide and remains a major challenge for clinicians and healthcare systems. It affects individuals across a wide range of ages, often leading to persistent disability, reduced quality of life and limitations in work and social participation [58–61]. The multifactorial nature of the condition, with contributions from biomechanical, psychological and lifestyle-related factors, complicates its management and explains the variable response to different therapeutic strategies [62–64]. Despite decades of research and the development of multiple treatment options, there is still no universal consensus on the most effective approach for all patients, and international recommendations remain inconsistent or lacking in detail regarding the prioritisation of specific physiotherapy modalities [65, 66]. Physiotherapy has consistently been regarded as a cornerstone of non-pharmacological care; yet, the diversity of available techniques and the heterogeneity of treatment protocols have fuelled an ongoing debate about which strategies provide the most meaningful and sustained clinical benefits [67, 68]. Education has also been increasingly incorporated into multimodal programmes.

According to the main findings of the present study, active physiotherapy was associated with greater outcomes compared with passive and the combination of passive and active physiotherapy in patients with mechanical and aspecific cLBP. The three treatment groups showed high baseline comparability under all evaluated parameters. The present study presents a head-to-head quantitative analysis of the considered outcomes of interest for the three categories being assessed in physiotherapy. In this study, the key assumptions of heterogeneity and transitivity were carefully considered. Although the included trials differed in terms of sample size and specific physiotherapy protocols, the random-effects model was applied to account for between-study variability, ensuring more conservative and reliable estimates. The transitivity assumption was supported by the comparable baseline characteristics and outcome definitions across studies, allowing valid indirect comparisons. Moreover, the global Wald test confirmed the overall consistency of the treatment network, and the inspection of funnel plots did not reveal major asymmetries suggestive of publication bias. Taken together, these elements support the robustness of the present findings despite the intrinsic variability of the available evidence.

The results obtained are in line with those of recent systematic reviews, which have observed very low to moderate evidence supporting the efficacy of exercise for managing LBP [69–71]. While including a larger number of patients, however, most previously published works considered a more heterogeneous population, including non-chronic LBP and patients both with and without radicular symptoms. Restricting the selection criteria of the present meta-analysis to a specific diagnosis allowed to analyse a more homogeneous patient cohort and thus obtain stronger evidence of the effects of active versus passive physiotherapy in the specific population of patients with chronic, mechanical or non-specific LBP.

Considering the association between trophism of the paraspinal muscles and LBP, the obtained results are not surprising. Patients suffering from cLBP showed atrophy of the multifidus and paraspinal muscles [72] and increased intramuscular fat infiltration [73–77]. Overall, patients with a diagnosis of LBP are also less likely to comply with the physical activity guidelines offered by the World Health Organization [78]. Interestingly, muscular atrophy has a stronger correlation to disability rather than with pain [79]; particularly in patients with mechanical cLBP, pain may be, at least in part, mediated by facet and disc degeneration. In this setting, active physiotherapy plays a central role in restoring mobility and strength, allowing patients to return to their activities of daily living with fewer restrictions. Further studies should focus on highlighting which specific domains of health-related quality of life can be best addressed with active and passive physiotherapy, thereby offering better counselling for patients.

While physical exercise can increase the trophism of the activated muscle, there is evidence (albeit low) that fatty degeneration is not reversible [80]. In this scenario, active physiotherapy may have the function of maintaining and preserving the non-degenerated muscular mass to limit the worsening of the symptoms, as inactivity is associated with higher muscular fatty degeneration and LBP [81].

It has been shown that a reduction in range of motion, and in particular the restriction of lateral flexion, is also associated with the development of LBP [82]. Both active and passive physiotherapy might help improve spine mobility, consequently improving the symptoms of cLBP. Exercise could also have an indirect effect mediated by the reduction of BMI. While obesity is associated with LBP [83], a lower BMI was correlated with better outcomes of exercise for LBP management [84].

While not as effective as active physiotherapy, passive management also leads to an improvement in pain symptoms and disability in patients with cLBP. This finding is consistent with previous data regarding the use of manipulation and mobilisation in this patient cohort [85]. Passive physiotherapy likely acts by improving spine mobility and releasing contraction and stretch-induced pain [35–38, 42], thus allowing patients to conduct activities of daily living with more ease. In particular, passive physiotherapy might be beneficial for patients with negative beliefs regarding active therapy concomitant with pain episodes. Overall, the efficacy of passive management alone in the setting of cLBP is still debated, and further studies will be required to strengthen the evidence supporting this management option [86].

It is important to note that the management of cLBP is becoming increasingly multimodal, involving the combination of various types of therapy such as physiotherapy, psychological management, acupuncture and pharmacological management [17, 68, 87–92]. While multimodal management surely represents an additional layer of complexity in evaluating the different strategies for treating cLBP, it also represents a further step towards patient-tailored management.

The presented study does not come without limitations. The main one is represented by the fact that the included studies differed in the type of active and passive physiotherapy offered and in the therapeutic regimen. Furthermore, the presence and type of concomitant pharmacological therapy also differed among the available works. However, the data were too heterogeneous and insufficient to allow a sub-analysis of these factors. Moreover, the included studies only showed a short-term follow-up. Future studies should investigate which type of active physiotherapy is most effective for the management of mechanical and non-specific cLBP and analyse the effects of physiotherapy on a longer follow-up and on the recurrence rate.

## Conclusions

Active physiotherapy showed better results than passive physiotherapy and a combination of both for the management of mechanical and/or non-specific, chronic LBP.

## Data Availability

The datasets generated during and/or analysed during the current study are available throughout the manuscript.
